# Hypereosinophilia With Organ Damage Secondary to Dupilumab Therapy: A Case Report and Literature Review

**DOI:** 10.1155/crdm/8570980

**Published:** 2026-10-04

**Authors:** Manar Alotaibi, Sadeem AlHelal, Noura Alotaibi, Fahad Alsudairy

**Affiliations:** ^1^ Department of Dermatology Prince Sultan Military Medical City Riyadh Saudi Arabia psmmc.med.sa; ^2^ College of Medicine Princess Nourah Bint Abdulrahman University Riyadh Saudi Arabia pnu.edu.sa

**Keywords:** atopic dermatitis, case report, dupilumab, hypereosinophilia, myocarditis

## Abstract

Atopic dermatitis (AD) is a chronic inflammatory skin condition characterized by pruritic, scaly, erythematous lesions, xerosis, and lichenification. Dupilumab, a monoclonal antibody targeting I‐ (IL)‐4 and IL‐13 signaling, is widely used to treat moderate to severe AD. We report a 22 year‐old male with a history of asthma and chronic childhood AD who was started on dupilumab 300 mg every 2 weeks after multiple relapses despite topical therapy. Baseline Eczema Area and Severity Index (EASI), Dermatology Life Quality Index (DLQI), and Physician Global Assessment (PGA) scores were not recorded; however, the patient showed marked clinical improvement of his skin disease, with a posttreatment EASI score of 1. Five months after dupilumab initiation, he presented with palpitations and pleuritic chest pain. His absolute eosinophil count was 2070 cells/μL, increased from a pretreatment baseline of 860 cells/μL, with an elevated Troponin I of 1.56 ng/mL. Electrocardiography showed normal sinus rhythm. Cardiac magnetic resonance demonstrated myocardial edema, early gadolinium enhancement, and late gadolinium enhancement involving the interventricular septum and adjacent walls, confirming myocarditis. A comprehensive workup, including antinuclear antibody, antineutrophil cytoplasmic antibody, renal function, a respiratory viral PCR panel, stool ova and parasites, and whole exome sequencing, excluded autoimmune, infectious, and familial causes. Computed tomography of the sinuses demonstrated moderate rhinosinusitis without invasive features. A diagnosis of eosinophilic myocarditis secondary to dupilumab‐associated hypereosinophilia was made. Dupilumab was discontinued, and the patient was treated with corticosteroids and colchicine, with resolution of eosinophilia and symptoms. Although dupilumab‐associated eosinophilia is usually mild and transient, this case highlights the importance of maintaining a high index of suspicion for rare but serious organ involvement, particularly in patients with new cardiopulmonary symptoms or baseline eosinophilia, while recognizing that routine eosinophil monitoring is not currently recommended for all patients receiving dupilumab.

## 1. Introduction

Atopic dermatitis (AD), also known as atopic eczema, is a chronic inflammatory skin condition characterized by pruritic scaly erythematous lesions, xerosis, and lichenification [1]. Its prevalence varies across regions, sex, and age group, affecting approximately 15%–20% of children and 2%‐3% of adults, with the highest rates observed in children under 1 year of age [2]. The prevalence of AD ranges between 3.5% and 4.9% in the United States, Canada, and Europe, with a slightly higher prevalence among female patients [3]. Clinically, infants under the age of two typically present with eczematous lesions involving the cheeks, scalp, and neck, whereas in adults, the neck, head, and flexural areas are more commonly affected [3].

Treatment strategies vary based on disease severity. Topical corticosteroids and calcineurin inhibitors (such as pimecrolimus or tacrolimus) form the mainstay of therapy to treat or prevent this condition [4], with mild cases often managed by emollients, anti‐inflammatory agents, and light therapy. However, moderate‐to‐severe AD frequently requires systemic therapy in addition to topical treatments [5].

Dupilumab, a monoclonal antibody that targets Interleukin (IL)‐4 and IL‐13 signaling pathways, is recommended for the treatment of moderate‐to‐severe AD [6]. A systematic review evaluating the safety and efficacy of dupilumab reported high completion rates and sustained clinical improvement across studies; the most prevalent adverse effects were conjunctivitis and allergic conjunctivitis [7]. Dupilumab has also been associated with elevations in blood eosinophil counts, particularly in patients with a higher baseline eosinophil level [7, 8]. Although the exact mechanism behind this eosinophilic response is not fully understood, it has been suggested that IL‐4 blockade leads to decreased vascular cell adhesion Molecule (VCAM)‐1 expression and reduced Eotaxin‐3 serum levels, therefore decreasing eosinophil adhesion to blood vessels and tissue migration, which in turn raises circulating eosinophil levels [8]. Although dupilumab‐associated eosinophilia is usually mild and transient, severe hypereosinophilia with organ involvement is rare but clinically important. The aim of this article is to report a rare case of hypereosinophilia with organ damage secondary to dupilumab therapy in a patient with AD.

## 2. Case Presentation

A 22‐year‐old male with a history of asthma presented to the dermatology clinic complaining of a chronic history of childhood AD with severe itchiness. Baseline Eczema Area and Severity Index (EASI), Dermatology Life Quality Index (DLQI), and Physician Global Assessment (PGA) scores were not recorded at presentation. Despite multiple repeated courses of topical steroids and calcineurin inhibitors with complete patient adherence to the medications, he had multiple recurrent relapses (Figure 1a–d). Therefore, considering the chronicity of his eczema, lack of response to topicals, and the distress it caused the patient, he was started on dupilumab therapy at a dose of 300 mg every 2 weeks. The patient showed marked clinical improvement of his skin disease on dupilumab, with an EASI score of 1 recorded prior to the onset of his cardiac symptoms.

**FIGURE 1:**
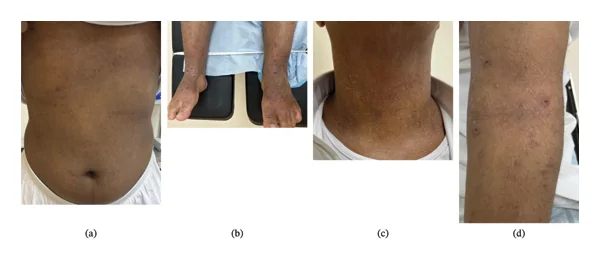
Atopic dermatitis: multiple ill‐defined lichenified plaques with overlying eroded nodules, lichenified papules, and excoriation marks over the trunk, neck, and antecubital fossa. (a) Trunk showing diffuse ill‐defined lichenified plaques. (b) Bilateral feet and ankles showing lichenified papules, eroded nodules, and excoriation marks. (c) Anterior neck showing lichenification. (d) Forearm/antecubital fossa showing excoriated lichenified papules.

Five months later, the patient was well until he presented to the emergency department with palpitations and chest pain that worsened with inspiration and improved while sitting forward. He denied fever, rash, or joint pain. His laboratory results revealed an absolute eosinophil count of 2070 cells/μL (reference range 0–400 cells/μL), increased from a baseline of 860 cells/μL before the initiation of dupilumab treatment. His Troponin I level was 1.56 ng/mL (reference range < 0.04 ng/mL), and his electrocardiogram (ECG) showed normal sinus rhythm (Figure 2). His chest X‐ray and echocardiography were normal, with an ejection fraction of 55% and no significant valvular disease.

**FIGURE 2:**
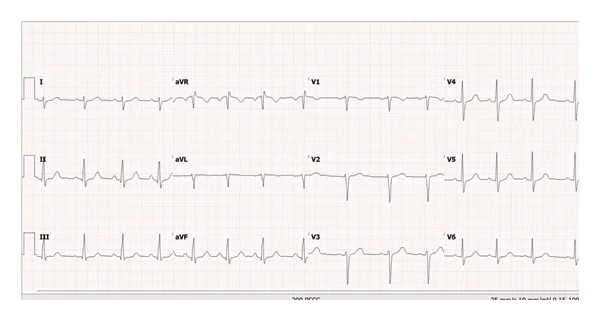
Twelve‐lead electrocardiogram obtained during the index presentation, demonstrating normal sinus rhythm.

Despite the normal echocardiogram, a cardiac magnetic resonance (CMR) was performed due to high clinical suspicion of myocarditis, which confirmed the diagnosis (Figure 3). Considering the patient’s CMR findings and eosinophil count, a diagnosis of eosinophilic myocarditis was made, and the patient was started on prednisolone 40 mg daily and colchicine 500 mcg daily. He was then investigated for his hypereosinophilia; he denied a prior history of shortness of breath, rash, joint pain, hemoptysis, oral ulcers, fever, weight loss, night sweats, or malaise and reported no new medications or recent travel.

**FIGURE 3:**
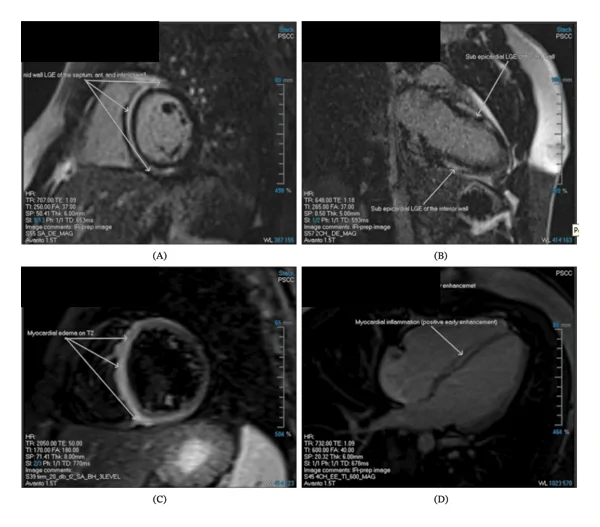
Cardiac magnetic resonance imaging findings consistent with myocarditis. (A) Late gadolinium enhancement (LGE), short‐axis view, showing a dense mid‐wall pattern involving the septum and the anterior and inferior walls. (B) LGE, two‐chamber long‐axis view, showing subepicardial enhancement of the anterior and inferior walls. (C) T2‐weighted imaging showing hyperintense signal at the mid‐interventricular septum, consistent with myocardial edema. (D) Early gadolinium enhancement demonstrating myocardial inflammation. The total manually drawn late‐enhancement area measured approximately 12 cm^2^; this pattern is in keeping with myocarditis rather than a myocardial infarction, given its noncoronary distribution.

Laboratory testing was negative for antinuclear antibody (ANA) and antineutrophil cytoplasmic antibodies (ANCA), and creatinine and blood urea nitrogen (BUN) were within normal limits. A respiratory viral PCR panel was negative for adenovirus, coronavirus, human metapneumovirus, human rhinovirus/enterovirus, influenza A, influenza B, parainfluenza virus, respiratory syncytial virus, and MERS‐CoV. Stool testing for ova and parasites was negative. Whole exome sequencing (WES) did not reveal an identifiable pathogenic mutation for familial eosinophilia syndromes. Computed tomography (CT) of the nasal sinuses demonstrated moderate rhinosinusitis without invasive features (Figure 4).

**FIGURE 4:**
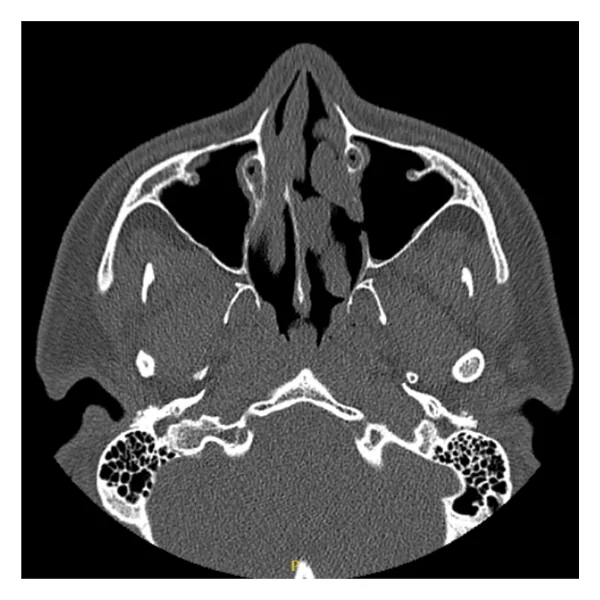
Axial computed tomography of the paranasal sinuses demonstrating moderate rhinosinusitis without invasive features.

The patient was referred to hematology‐oncology, who diagnosed reactive or secondary eosinophilia. Dupilumab was discontinued, which resulted in normalization of the eosinophil count (190 cells/μL) and resolution of symptoms. However, during follow‐up, his skin began to flare again, though this was controlled with topical therapy alone. His eosinophil count was monitored at subsequent visits and increased mildly to 790–850 cells/μL, which was considered his eosinophilic baseline; no further intervention was undertaken, as he remained clinically well.

## 3. Discussion

This case highlights a rare instance of dupilumab‐associated hypereosinophilia complicated by eosinophilic myocarditis in a patient treated for AD. Although eosinophilia is a well‐recognized finding in patients receiving dupilumab, progression to hypereosinophilia with clinically significant end‐organ involvement remains uncommon. Across clinical trials and long‐term safety data, conjunctivitis remains the most prevalent adverse effect associated with dupilumab, whereas severe eosinophilia associated with organ damage has mainly been identified in postmarketing reports [9, 10].

In our patient, the eosinophil count rose from 860 cells/μL at baseline to over 2000 cells/μL after five months of therapy, accompanied by chest pain, palpitations, and a markedly elevated Troponin I level. This latency aligns with other published reports describing eosinophil‐related symptoms beginning within 2–6 months of dupilumab initiation. Reported manifestations have ranged from eosinophilic pneumonia and worsening sinus disease to the less frequent but more serious eosinophilic myocarditis [11, 12].

Beyond the individual cases described above, dupilumab‐associated eosinophilic organ complications have increasingly been recognized across a range of indications. Two recent evidence syntheses have helped consolidate this literature. A systematic review by Lazzeroni et al. pooled 35 reports describing 53 patients with dupilumab‐associated eosinophilic complications, most commonly eosinophilic granulomatosis with polyangiitis (24 patients), eosinophilic pneumonia (15 patients), and hypereosinophilic syndrome (6 patients); complications developed after a median of 9 weeks, with a median eosinophil count of 6.38 × 10^9^ cells/L at diagnosis, dupilumab was discontinued in 89% of cases, and no deaths were reported among the published cases [13]. A separate narrative review and pharmacovigilance analysis by Suter et al. identified a comparable set of previously reported patients with eosinophilic organ complications (predominantly eosinophilic pneumonia, eosinophilic granulomatosis with polyangiitis, and hypereosinophilic syndrome) and found disproportionate safety signals for these conditions, as well as for eosinophilic myocarditis specifically, within the WHO VigiBase pharmacovigilance database [14]. Isolated case reports of acute eosinophilic myocarditis occurring shortly after dupilumab initiation, in patients without preexisting cardiac disease, have also been published and support the mechanistic plausibility of the presentation described here [15]. Taken together, these reports support the hypothesis that IL‐4/IL‐13 blockade may, in a small subset of patients, prolong eosinophil survival in the circulation rather than uniformly suppressing Type 2 inflammation, although several reported EGPA cases may alternatively represent unmasking of preexisting eosinophilic vasculitis during systemic corticosteroid tapering rather than a direct drug effect.

The immunologic basis for these reactions remains incompletely understood. Under normal conditions, IL‐4, IL‐5, and IL‐13 drive Type 2 inflammation and regulate eosinophil trafficking, activation, and survival; dupilumab blocks IL‐4 and IL‐13 signaling [16–19]. However, several studies suggest that this blockade indirectly influences IL‐5 pathways, affecting eosinophil survival and delaying their clearance. This may explain why many patients experience asymptomatic eosinophilia, whereas a few develop eosinophil‐mediated tissue damage [19, 20]. Previous literature has proposed downregulation of VCAM‐1 and the surface markers CD49d and CXCR4 on eosinophils as a possible explanation for dupilumab‐induced eosinophilia [19]. Hamilton et al. further found that dupilumab suppressed most Type 2 inflammatory biomarker levels across different patient groups [20].

In our patient, CMR demonstrated a Hyperintense T2 signal at the mid‐interventricular septum, in keeping with myocardial edema; positive early gadolinium enhancement of the interventricular septum, indicating myocardial inflammation, and a dense linear mid‐wall late gadolinium enhancement (LGE) pattern that became subepicardial in some segments, involving the mid‐interventricular septum and extending to adjacent segments of the anterior and inferior walls, with a manually drawn total enhancement area of approximately 12 cm^2^ (Figure 3). These findings fulfilled the updated Lake Louise criteria for myocarditis and were not consistent with a myocardial infarction pattern, given the noncoronary distribution of the LGE. This case reinforces the value of CMR as a highly sensitive, noninvasive tool for identifying myocardial inflammation and fibrosis, particularly when performed early and analyzed with appropriate tissue‐characterization sequences [21, 22].

Management approaches to dupilumab‐associated hypereosinophilia vary according to severity. Most cases improve with discontinuation of dupilumab and initiation of systemic corticosteroids, as observed in our patient. For refractory or steroid‐dependent cases, antieosinophil biologics such as mepolizumab or benralizumab (anti‐IL‐5 or anti‐IL‐5R agents) have been used successfully, including a previously reported case of acute myocarditis following dupilumab initiation [15, 23]. Close collaboration between cardiology, dermatology, and immunology specialists is essential to weigh the risks and benefits of continuing or discontinuing dupilumab and to consider alternative therapies for AD control [23].

We acknowledge that current guidelines (including those from NICE, the European Academy of Dermatology and Venereology [EADV], the American Academy of Dermatology [AAD], and the manufacturer’s product information) do not recommend routine blood eosinophil monitoring for all patients receiving dupilumab, since transient elevations are common, and the vast majority is clinically insignificant [9, 24]. Kemp et al. proposed a more intensive monitoring schedule specifically in the context of chronic rhinosinusitis with nasal polyps (eosinophil counts at baseline, week 4, and every 12 weeks thereafter, with more frequent monitoring for counts ≥ 3000 cells/μL and treatment discontinuation at ≥ 5000 cells/μL [25]) which has not been adopted into general dupilumab guidelines. Based on our case and the reviewed literature, we suggest that closer clinical and laboratory evaluation may be reasonable in a targeted subset of patients (specifically those with baseline eosinophilia or those who develop new cardiopulmonary symptoms such as chest pain or dyspnea) rather than as a universal monitoring protocol. Eosinophil testing should otherwise be reserved for patients with clinically indicated symptoms, consistent with current guideline recommendations.

With regard to potential regional or population‐based differences in dupilumab‐associated eosinophilia, we are not aware of a study that directly compares Asian, European, and North American cohorts using matched methodology. Available real‐world eosinophilia rates vary considerably across cohorts and inflammatory indications rather than by region alone: reported hypereosinophilia rates range from 8.7% to 16.2% in Western asthma and chronic rhinosinusitis with nasal polyps populations [12, 25], and up to 40% in a small Chinese cohort with severe asthma [26], while Japanese postmarketing surveillance in AD reported eosinophilia in only 0.5% of patients, likely reflecting the lower baseline eosinophil burden of AD relative to asthma or CRSwNP [27]. A separate real‐life study from Italy reported hypereosinophilia in 15.15% of patients treated for Type 2 diseases, none of whom required discontinuation for organ‐related complications [28]. Background epidemiological data further suggest that chronic rhinosinusitis with nasal polyps in Western populations is predominantly eosinophil‐driven, whereas East Asian populations more frequently display a mixed or noneosinophilic endotype [29], which may partly explain regional variation in baseline eosinophil counts and comorbidity patterns, though a genetic contribution has also been proposed. Given the absence of dedicated comparative data, we present these observations as hypothesis‐generating context rather than firm conclusions, and we highlight this as an area warranting prospective, multiregional study.

This case report has several limitations. A single case report cannot establish causality between dupilumab and myocarditis. Other recognized causes of myocarditis were considered, including viral infection and recent vaccination. A respiratory viral PCR panel was negative for adenovirus, coronavirus, human metapneumovirus, human rhinovirus/enterovirus, influenza A and B, parainfluenza virus, respiratory syncytial virus, and MERS‐CoV; Coxsackievirus, herpes simplex virus, Epstein–Barr virus, and recent mRNA vaccination were not specifically tested for but were considered clinically unlikely given the absence of a viral prodrome or recent vaccination and the close temporal relationship between the rise in eosinophil count and the onset of myocarditis. Taken together with the negative ANCA/ANA and the rapid normalization of eosinophil counts after discontinuing dupilumab, a drug‐associated hypereosinophilic process remains the most likely explanation. Reporting such cases in clinical practice is an effective means of identifying serious postmarketing adverse events and informing future monitoring guidance.

In summary, AD is a common chronic inflammatory skin disorder managed with topical and systemic therapies, and dupilumab has significantly improved outcomes in moderate‐to‐severe cases. Although dupilumab‐associated eosinophilia is typically mild and transient, clinicians should remain alert to the rare possibility of eosinophilic organ damage, particularly in patients with baseline eosinophilia or new cardiopulmonary symptoms, and should pursue prompt multidisciplinary evaluation when such features arise.

## Funding

No funding was received for this research.

## Consent

Written informed consent was obtained from the patient for publication of this case report and accompanying images.

## Conflicts of Interest

The authors declare no conflicts of interest.

## Patient Perspective

The patient reported significant improvement in symptoms after discontinuation of dupilumab and expressed satisfaction with the management plan.

## Data Availability

The data that support the findings of this study are available on request from the corresponding author. The data are not publicly available due to privacy or ethical restrictions.
